# Neural connectivity, music, and movement: a response to Pat Amos

**DOI:** 10.3389/fnint.2013.00029

**Published:** 2013-04-24

**Authors:** Eric Barnhill

**Affiliations:** ^1^Department of Music, Edinburgh College of Art, Institute for Music in Human and Social Development, The University of EdinburghEdinburgh, UK; ^2^Clinical Research Imaging Centre, School of Clinical Sciences and Community Health, College of Medicine and Veterinary Medicine, The University of EdinburghEdinburgh, UK

## Where rhythms coincide?

Pat Amos documents the power of the rhythmic moment in autism, connects it to current thinking in developmental psychology, and draws practical lessons for therapeutic intervention. One question therapists in practice may struggle with is convincing some parents and other professionals of the potential power of these types of interventions. As a movement and music therapist I recall a mother telling me about her recent visit to a prominent pediatric neuropsychiatrist. The neuropsychiatrist was once again recommending a regimen of heavy medication and behavioral therapy. The mother told her that she felt her son was making great progress through work with rhythm and movement. “That won't hold,” said the neuropsychiatrist.

Will it hold? Can a movement based intervention compete with a pharmaceutical one? The idea can meet with great skepticism. However, there is a strong argument to be made from neurobiological theory that a rhythmic intervention holds the potential to be at least as powerful as a chemical intervention, and the broader one's investigation into neurobiology, the more the arguments for this view accumulate.

## The evolutionary view: brain as rhythmically-driven predictor of movement

The ability of rhythm and music to empower those suffering from delay, disorder or degeneration has been amply documented by therapists and researchers [among many (Sacks, 2008)]. Similarly, our understanding of neural activity as a rhythmic phenomenon, from the single-neuron motor pattern generation in *Clione* (Satterlie, 1985) to the recurrent thalamocortical resonance that supports human consciousness (Buzsáki and Draguhn, 2004), is equally well established. Physiological and musical rhythms are qualitatively distinct, but intersect in body movement. All musical performance, recent breakthroughs in mind-machine interface aside [such as (Miranda, 2006)], is movement, and our preferred modes of interacting with music, despite a propensity for studying passive listening in the lab out of convenience, are almost entirely physically active (Blacking, 1973; Small, 1998). Our movements, of course, are generated physiologically, causing a musical movement to by nature be an interaction of the two. It is feasible, then, to describe an interaction between musical and neural rhythms so long as it is understood as an embodied event.

As Amos points out, dynamic systems are increasingly used as models for life at nearly all scales, including human development and human consciousness. An even broader view places life as a subspecies of dynamic systems, or as a recent article in Cell puts it, “we biologists are studying what are probably the world's most interesting nonlinear dynamical systems” (Ferrell et al., 2011).

In an energetically closed system energy dissipates in accordance with the second law of thermodynamics, resulting in an increase in entropy or disorder (Fermi, 1956). In the presence of a stream of energy, however, systems often grow in their efficiency of dissipation by becoming more ordered (Nicolis and Prigogine, 1977). This leads to spontaneous order, the hallmark of dynamic systems, in a thermodynamically open environment (Varela et al., 1974; Haken, 1980). Steady states have limited resiliency in the face of perturbation, and as a result the most stable spontaneously ordered systems show oscillatory behaviors (Haken, 1980; Kelso, 1995).

Even in the most primitive organisms life is an oscillatory dance between a supercritical, energy-releasing core and a subcritical, energy dampening boundary (Kauffman, 1996). In multicellular organisms the need for coordination grows much more complex, and “oscillation-based synchrony is the most energy-efficient physical mechanism for temporal coordination” (Buzsáki and Draguhn, 2004). On the evolutionary time scale, movement develops, followed by senses to guide the movement; a means of communication is needed between the two, and the most efficient means, electricity, wins out: the neuron (Llinás, 2002). Neural networks do not issue serial commands but self-organize into oscillatory states, whether the simple wing flapping of *Clione Limacina* (Satterlie, 1985) or the complex networks recruited for human ambulation (Prentice et al., 1998; Ijspeert, 2008).

As animals grow in size and sophistication, the nervous system develops the interneuron, allowing communication between sense and movement to be modulated (Llinás, 2002). Massive interneuron growth gives rise to the brain and of what is thought to be the essential function of the brain: prediction of movement. Multiple strains of neuroscience have converged on this same idea: for example, neurobiologist Rodolfo Llinàs states that “The capacity to predict the outcome of future events—critical to successful movement—is, most likely, the ultimate and most common of all global brain function” (Llinás, 2002), while neuropsychologist Alain Berthoz writes that “the brain is a biological simulator that predicts by drawing on memory and making assumptions” (Berthoz, 2000) and neurophysiologist Gyorgy Buzsàki writes that “brains are foretelling devices and their predictive powers emerge from the various rhythms they perpetually generate” (Buzsaki, 2009) The ability to link human brain waves to specific types of content is of course the basis of neurofeedback (Cantor, 1999).

## White matter, cortical connectivity, and multimodality

This rhythmic perspective is worth keeping in mind when investigating the booming recent literature on white matter connectivity, made possible through advances in diffusion weighted imaging. The brain's white matter tracts connect regions of the cortex to each other as well as to sensory regions via the gateway of the thalamus (Kandel et al., 2000). A symphony of thalamocortical oscillations passes along these channels, ranging in frequency from infra-slow to ultra-fast (Steriade et al., 1995). Divergent development of white matter has been found at under a year of age in children who later develop an ASD diagnosis (Wolff et al., 2012). Across the lifespan, the white matter of individuals on the autism spectrum is characterized as less organized and less well connected (a variety of variables are assembled to determine this such as less fractional anisotropy and greater radial diffusivity) (Travers et al., 2012). One finds in these recent white matter studies a compelling structural analogue to Amos' descriptions of autism as connectivity-related impairment affecting cross-modal processing, resulting in a signal that is at some point “scrambled.”

Worth noting in our rhythmic context, however, is that the number of distant neuronal connections in the brain is quite small compared to the local ones even in a healthy brain, as oscillatory synchrony represents a flexible and energy-efficient alternative to hard wiring in the communication of distant cortical regions (Buzsáki and Draguhn, 2004; Schnitzler and Gross, 2005). We can therefore think of the brain as having dual, deeply entwined connectivities—one architectural and one rhythmic. Of the two, it is the oscillatory that appears to be both more flexible and more thermodynamically efficient, and may represent the greater portion of the brain's connectivity.

Amos cites a wide array of evidence-based therapists who use “rhythm and timing as scaffolding to build social and communicative interactions.” An intriguing hypothesis from the standpoint of neural science is whether, given an impairment in structural connectivity, the more dynamic connectivity of rhythmic oscillation can make up the difference. In this case, the rhythm is almost literally “scaffolding” the disordered white matter, providing structure and connectivity in the absense of its usual biological substrate.

## Functional connectivity and a “dual connectivity” hypothesis

The hypothesis advanced here is that one form of connectivity—oscillatory synchrony—might be able to make up for disruption in another form of connectivity, the structural connectivity of white matter tracts. This could be part of what explains the often magical-seeming powers of music to enable the disabled, whether in motor or social domains. Longer-distance oscilatory networks can be created through the synchronized resonance of local brain pathways, allowing multimodal information to communicate through an alternate route.

A test of such a hypothesis could include coordinated diffusion-weighted imaging studies and fMRI or EEG functional connectivity studies. A review (Schipul et al., 2011) notes the consistent findings of functional underconnectivity among diverse brain regions in autistic subjects versus controls, in both task-dependent and resting state conditions. If musical movement could aide the brain in its ability to rhythmically coordinate, this may express itself in an increase of functional connectivity relative to structural connectivity during a condition of active music making or rhythmic movement.

Beyond a present musical stimulus, could work with music have a more lasting effect on the brain's ability to coordinate diverse brain regions and sensory modes, that is to say, could the “scaffolding” effect of a rhythmic or musical intervention have neuroplastic impact? While a neuroplastic effect might express itself as a structural-connectivity independent functional-connectivity increase, evaluation methods would have to contend with the abundant evidence for white matter plasticity in general (Jäncke, 2009), and white matter plasticity in response to musical therapies in particular (Schlaug et al., 2009). It would be difficult to predict whether functional or structural connectivity would change together, separately, or on different but related time courses.

## Practical neuroplasticity: assessment of movement

While connectivity studies might provide a compelling evidence of a mechanism for the power of music, they will not indicate a practical pathway to implement it. For that, the principal mode of interaction between musical and physiological rhythms must be returned to: movement.

It was my experience as therapist that tapping the true neuroplastic potential of music and rhythm requires incorporating more powerful tools of movement analysis and movement work. This is why, though my original background is studying music, I trained in movement methods in order to best incorporate music as therapy, and described my own therapy work, Cognitive Eurhythmics, as a movement therapy that incorporated rhythm and music.

The movements of the body are not simply a vehicle for transmitting music to the brain; the muscles and bones are the true domain where music and physiology come together. With a trained eye for functional movement, it is possible to see the way a particular person's movement does or does not reflect music, and over time, to see the movements grow more musical. Over time movement patterns that emerge from representing music can be redirected into function real-life behaviors.

Musicality of movement can be analysed by many different approaches; movement could be investigated for the harmonious interaction of body, shape, space and effort, as in Laban Movement Analysis (von Laban, 1967; Bartenieff, 1980); the relation of distal to proximal effort, the integration of the movement through the body, and the amount of parasitic movement, as might be done using the Feldenkrais Method (Feldenkrais, 1980; Rywerant, 1983) or the relationship of time, space, and energy, as in the Dalcroze method (Jaques-Dalcroze, 1921; Dutoit, 1971). It is one thing to play slow music for a child and watch the child slow down with it, often an accomplishment in itself. But how is the weight transferring over the foot? How well are the head and eyes integrating with the locomotion? How reversible is the movement and how ballistic? These are real-time questions that can rapidly empower the development of new behavior patterns in a therapy session. By applying these tools to interactions of music and movement, a course of improvment can be charted, gradually empowering a student until they can use their own inner rhythmic faculties to master previously insurmountable problems.

The challenge is that these methods take years of training, as the instructor must learn good movement from the inside out, in order to have a practical eye able to assess the movement in others. However, without these tools, the most powerful part of a therapy session—the quality of the movement—is not being tapped for its true potential.

In her summary of rhythm and timing in autism, Amos has documented well the psychological case for dancing with autism. Such a case has strong theoretical support from evolutionary neurobiology. Neurophysiology and neuroimaging together suggest a “dual connectivity” model that could provide a mechanism for the documented power of music, and this idea can be empirically investigated by testing for a divergence between functional and structural activity under condition of active music making. Finally, given the embodied nature of the music-physiology interaction, trained practitioners of sophisticated movement analysis and training methods like Laban, Feldenkrais, and Dalcroze could be tapped to develop a new generation of powerful movement-and-music based therapeutic practices.
